# Cocaine addicted rats show reduced neural activity as revealed by manganese-enhanced MRI

**DOI:** 10.1038/s41598-020-76182-3

**Published:** 2020-11-09

**Authors:** Nazzareno Cannella, Alejandro Cosa-Linan, Tatiane Takahashi, Wolfgang Weber-Fahr, Rainer Spanagel

**Affiliations:** 1 Institute of Psychopharmacology, Central Institute of Mental Health, Medical Faculty Mannheim, Heidelberg University, Mannheim, Germany; 2 Research Group Translational Imaging, Department of Neuroimaging, Central Institute of Mental Health, Medical Faculty Mannheim, Heidelberg University, Mannheim, Germany

**Keywords:** Neuroscience, Diseases of the nervous system, Addiction

## Abstract

Cocaine addiction develops as a continuum from recreational to habitual and ultimately compulsive drug use. Cocaine addicts show reduced brain activity. However, it is not clear if this condition results from individual predisposing traits or is the result of chronic cocaine intake. A translational neuroimaging approach with an animal model distinguishing non-addict-like vs. addict-like animals may help overcome the limitations of clinical research by comparing controlled experimental conditions that are impossible to obtain in humans. Here we aimed to evaluate neuronal activity in freely moving rats by manganese enhanced magnetic resonance imaging in the 0/3crit model of cocaine addiction. We show that addict-like rats exhibit reduced neuronal activity compared to cocaine-naïve controls during the first week of abstinence. In contrast, cocaine-experienced non-addict-like rats maintained their brain activity at a level comparable to cocaine-naïve controls. We also evaluated brain activity during cocaine bingeing, finding a general reduction of brain activity in cocaine experienced rats independent of an addiction-like phenotype. These findings indicate that brain hypoactivity in cocaine addiction is associated with the development of compulsive use rather than the amount of cocaine consumed, and may be used as a potential biomarker for addiction that clearly distinguishes non-addict-like vs addict-like cocaine use.

## Introduction

Cocaine addiction is a chronic multi-dimensional relapsing disorder affecting 15–20% of the total of regular cocaine users^1–3^. The DSM5-based dimensional diagnostic approach defines cocaine addiction as a continuum from recreational drug use to habitual and ultimately addicted behavior mainly characterized by loss of control and frequent relapses. Loss of control and relapse are associated with altered brain functions as revealed by several neuroimaging techniques. Specifically, a hypoactive brain state in addicted patients compared to healthy control has been demonstrated by functional neuroimaging studies^4,5^. In fact, brain hypoactivity is a suggested biomarker of cocaine addiction as it is associated with reduced ability to experience reward^6,7^ and reduced ability to control impulses^8–10^. Brain hypoactivity also predicts relapse in abstinent patients and is as such seen as a determinant of relapse risk^11–13^. However, it is not clear if reduced brain activity in cocaine addicts is causally involved in mediating core symptoms of addictive behavior, derives from individual predisposing traits, or is simply the result of chronic cocaine intake. On the clinical level, it is difficult to determine which one of these alternative conclusions is correct as it is very challenging to include a cocaine experienced control group with no diagnostic criteria of cocaine addiction but comparable levels of drug exposure and similar quality of life conditions as cocaine addicted patients.

Here, a translational neuroimaging approach with an animal model in which non-addict-like vs. addict-like animals can be distinguished could be helpful to overcome the limitations of clinical research by comparing controlled experimental conditions impossible to obtain in humans. Therefore, we used the DSM-based multi-dimensional 0/3 criteria (0/3crit) rat model of cocaine addiction^14–17^ to study differences in brain activity between addict-like (3crit), non-addict-like (0crit) and cocaine-naïve age-matched control rats. Similar to prevalence rates of cocaine addicted individuals^1–3,18^, the 0/3crit model of addiction results in 15–20% of a rat cohort losing control over cocaine-seeking and intake after prolonged training (characterized as 3crit rats), whereas a plurality (30–40%) of animals maintain control over cocaine (0crit rats). Importantly, individual differences between subjects showing addict-like and non-addict-like behavior are independent of differences in cocaine intake in this model. In other words, despite the fact that 0crit and 3crit rats consume similar amounts of cocaine over time, 3crit rats show clear distinct features of addiction-like behavior, including (i) the inability to refrain from drug seeking, (ii) high motivation for the drug, and (iii) maintained drug use despite negative consequences^15^. In a previous study we applied [^18^F]-fluorodeoxyglucose-positron emission tomography (FDG-PET) to the multi-dimensional 0/3crit model of cocaine addiction, and showed reduced cortical glucose metabolism in 3crit (cocaine addict-like) rats as compared to 0crit (non-addict-like) rats and age-matched cocaine-naive rats^19^. This previous molecular neuroimaging approach demonstrates the translatability of this model to the human situation, as cocaine addicted patients also exhibit reduced cortical glucose metabolism^5,20,21^.

In the present study we aimed to evaluate neural activity in freely moving rats throughout a period of cocaine abstinence by manganese enhanced magnetic resonance imaging (MEMRI) in rats subjected to the 0/3crit model of cocaine addiction. MEMRI uses the paramagnetic properties of manganese (Mn^2+^) to trace anterograde axonal transport and neural activation. Mn^2+^ is a Ca^2+^ analog that enters excitable cells via voltage-gated calcium channels and accumulates in mitochondria via the mitochondrial Ca^2+^ uniporter. Mn^2+^ accumulated in the brain shortens the T1 relaxation times of water, yielding enhanced signal in spin lattice relaxation time T1-weighted images. Due to this paramagnetic property of Mn^2+^, calcium dependent neural activity can be tracked by in vivo and non-invasive MRI^22^. One major advantage of MEMRI is that different experimental and disease conditions can be studied in behaving rats, whereas other animal-based functional MRI techniques usually require immobilized or anaesthetized animals^23^. MEMRI has previously been used to study brain-wide activation pathways induced by a cocaine challenge, cocaine-induced behavioral sensitization, and sex differences in cocaine responses^24–26^. MEMRI has also been used to study brain activity associated with acute nicotine withdrawal^27^, oxycodone induced neural activation^28^, and brain activity during voluntary alcohol drinking^29^.

Abstinent cocaine-dependent subjects show altered brain activity^5,9,13,21,30–34^. However, the intrinsic limitations of clinical studies make it difficult to understand whether this condition is a mere consequence of massive cocaine consumption or is associated to the patients’ psychiatric profile independently of the quantity of cocaine consumed. Therefore we present a study using MEMRI to measure brain activity during early cocaine abstinence in freely-behaving addict-like (3crit) and non-addict-like (0crit) rats that consumed similar amount of cocaine in their lifespan and were subjected to identical experimental conditions. In addition, as relapse to binge intoxication after abstinence is a hallmark of addiction^35–37^, we also exploited MEMRI to measure brain activity during a binge cocaine intoxication session in addict-like and non-addict-like rats after a period of withdrawal.

## Methods

Experimental procedures were performed in accordance with the NIH ethical guidelines for the care and use of laboratory animals and the EU Directive 2010/63/EU for animal experiments, and were approved by the local animal care committee (Regierungspräsidium Karlsruhe, Germany).

### Subjects

The rats used for MEMRI assessment are part of a previous multi-modal neuroimaging study in which we examined the relationship between grey matter volume and cocaine addiction subdimensions^38^. Non-addict-like (0crit, N = 10), addict-like (3crit, N = 9) and age-matched cocaine-naïve control (AgeControl, N = 10) rats were subjected to MRI acquisition^38^.

### Behavioral characterization

Male Sprague–Dawley (n = 71) rats were single-housed in a 12 h/12 h reversed cycle (temperature, 22 ± 1 °C; humidity 60 ± 5%). They were fed with 20 g/d of standard chow food and had water available ad libitum. Ten rats were randomly assigned to the age-matched cocaine naïve control group (AgeControl) and subjected to sham procedures; sixty-one rats underwent cocaine self-administration (CSA) training and tests. Behavioral protocols were similar to those previously described by us and others^14,15,17,19,38–42^. Briefly, behavioral training and tests were run in self-administration boxes (Imetronic, France) equipped with two nosepoke holes, one designated as the active hole and associated with cocaine delivery and the other designated as the inactive hole. Self-administration boxes were further equipped with a white cue light above each hole, a green cue-light on the left of the active hole, a blue cue light on the wall opposite to the active hole, and a house-light on the ceiling. CSA sessions consisted of three 40-min drug-periods alternated by two 15-min no drug-periods. During drug periods, signaled by the illumination of the blue light, nose poking in the active hole under a fixed ratio 5 (FR5) schedule of reinforcement delivered an infusion of 0.8 mg/kg of cocaine and illuminated the cue-light positioned above the hole for five seconds. Each infusion was followed by a 40-s time out during which active nosepokes were not reinforced. During no drug periods the blue cue light was extinguished, the house light was illuminated, and active nosepokes were recorded but not reinforced. The 0/3crit model of addiction adopted here is intended to study individual predisposition to show addiction-like behavior independent of the amount of cocaine consumed^15,16^. Therefore, in line with our previous studies^17,19^, a maximum of 35 infusions per session was allowed during SA training. Sessions were run five days a week. After forty-four CSA training sessions, three addiction-like criteria were scored in each rat. The first criterion, *persistence in drug-seeking* when the drug is signaled as not available, was measured by the number of active nosepokes during the no drug periods averaged over the last three days of CSA training. The second criterion, *motivation for cocaine*, was measured by the break point reached in a CSA session under a progressive ratio schedule of reinforcement. This test was performed on session forty-five under environmental conditions similar to drug periods, except that the responses to the active hole necessary to obtain a cocaine infusion was increased after each infusion according to the following schedule: 10, 20, 30, 45, 65, 85, 115, 145, 185, 225, 275, 325, 385, 445, 515, 585, 665, 745, 835, 925, 1025, 1125, 1235, 1345, 1465, 1585, 1715, 1845, 1985, 2125, 2275, 2425, 2585, 2745, 2915, 3085, 3265, 3445, 3635, 3825, 4025, 4225. The last ratio completed was defined as the break point (BP) and used to score motivation for cocaine. The PR session stopped after 5 h or if 1 h elapsed since the last infusion earned. Rats were then subjected to four baseline CSA sessions and on session fifty the third addiction criterion, *resistance to punishment*, was tested. This criterion was scored by the maintenance of cocaine use when drug-seeking and taking is punished by an electric foot-shock. The session lasted 40-min and was a modification of the first drug-period. Cocaine infusions were delivered under an FR5 schedule; however, after the first nosepoke the green cue-light was illuminated to signal the presence of the foot-shock, the fourth nosepoke resulted in a foot-shock (0.2 mA for 1 s), and the fifth nosepoke resulted in both a cocaine infusion and a foot-shock. Rats had 1 min to produce the first 4 pokes and then 1 min to produce the fifth poke; if these requirements were not met, the green cue light extinguished and the sequence reinitiated.

#### Characterization of addiction-like behavior

In each of the three criteria, rats scoring above the 60th percentile^16,17,19,38,42^ of the distribution were defined as positive; therefore, based on the number of positive criteria met, rats were allocated into four groups 0crit, 1crit, 2crit and 3crit. 0crit and 3crit rats, defined as non-addict-like and addict-like respectively, were selected for MRI experiments.

#### Ad-libitum CSA test

As explained above, during CSA training a limit of 35 infusions per session was imposed to highlight individual predisposition to addiction independent of the quantity of cocaine consumed. However, increased drug consumption is a trait associated with addiction-like behavior^15,43^. Therefore, after 0/3crit characterization, rats selected for MRI experiments were subjected to four additional CSA baseline training sessions before being tested in an ad-libitum cocaine test. The *ad-libitum* CSA session was identical to a drug-period described for baseline CSA except that it lasted 2 h and no limit to the number of infusions earned was imposed.

After *ad-libitum* testing, rats underwent between five and eleven CSA baseline sessions before the first MRI scanning was acquired. Specifically, after the fifth baseline session, every day, five rats (counterbalanced across groups) were randomly selected and maintained in abstinence for two days to prevent the acute pharmacological effects of cocaine from affecting MRI signals. On the third day of abstinence MRI scanning was acquired.

### Experiment 1: Neuronal activity during early cocaine abstinence evaluated by Mn^2+^ accumulation

Two days after the last session of the cocaine self-administration (CSA) training as described above and in^38^, rats underwent Mn^2+^ free MRI acquisition. We chose this time point to avoid the influence of acute systemic cocaine on MRI signals. Manganese administration began 24 h after Mn^2+^ free MRI acquisition. MnCl_2_ × 2H_2_O was dissolved in saline (pH adjusted to 7.4) and administered subcutaneously by osmotic mini-pumps (model 2001, Alzet, USA) at the speed of 1 μl/hour over four days. Each rat received a total MnCl_2_ dose of 120 mg/kg. The concentration of MnCl_2_ in the pumps was adjusted according to body weight. Prior to implantation, the pumps were primed in a 37 °C saline solution. Mini-pumps where implanted subcutaneously on the rat dorsum under isoflurane anesthesia. After completion of MnCl_2_ administration (i.e. five days after Mn^2+^ free MRI, corresponding to seven days of cocaine abstinence), a new MRI scanning was acquired, this time with Mn^2+^ on-board. Mini-pumps were removed from anesthetized rats immediately before Mn^2+^ on-board MRI acquisition (Fig. 2A).

### Effect of Mn^2+^ treatment on rats’ behavior and health

In order to control for possible behavioral impairments due to Mn^2+^ treatment, we monitored daily water and food consumption, home-cage locomotor activity and rats’ body weight during the Mn^2+^ infusion period.

Home-cage locomotor activity was monitored by an infrared sensor connected to a recording and data-storing device (Mouse-E-Motion by Infra-e-motion, Henstedt-Ulzburg, Germany) as we previously described^44^. Briefly, the device was fixed above the cage 30 cm from the bottom, so that the rat was detected at any position inside the cage. The device sampled every second. The sensor detected body movement of the rat of at least 1.5 cm from one sample point to the successive one. The data collected by each Mouse-E-Motion device were downloaded into a personal computer and processed with Microsoft-Excel. Monitoring of locomotor activity started 3 days before minipump implantation and continued until the day after Mn^2+^ on-board MRI acquisition.

We also determined whether Mn^2+^ treatment impaired cocaine reinforcement. Twenty-four hours after Mn^2+^ on-board MRI acquisition, rats were subjected to a two-hours *ad-libitum* CSA session as described above.

### Experiment 2: Neuronal activity during 24 h binge CSA session evaluated by Mn^2+^ accumulation

One month after termination of the CSA training, i.e. after a three-week washout period from the Mn^2+^ onboard acquisition of experiment 1 (Fig. 2A), another Mn^2+^ free T1 acquisition was acquired. At this time point Mn^2+^ induced enhancement is expected to have declined to baseline-comparable level^45^. One week later, rats received a sub-cutaneous treatment with 80 mg/kg of MnCl_2_ × 2H_2_O before undergoing a 24 hour-binge CSA session. In order to deal with toxicity associated with high acute Mn^2+^ load, we divided the 80 mg/kg dose in three sub-injections separated each other by 1 h, which resulted in sub-doses having none or minimal impact on behavior^46^. Each sub-dose was delivered in a volume of 5 ml/kg. CSA session started immediately after the third sub-treatment, i.e. 2 h after the first MnCl_2_ × 2H_2_O injection. This timing allowed us to start CSA after a 2 h time-window where scarce quantity of Mn^2+^ is detected in the brain parenchyma^45^ and therefore Mn^2+^ uptake would reflect exclusively brain activity during the 24 h CSA session. Self-administration schedule was identical to the 2 h session described above, except that—to reduce overdose risk—time-out period was increased to three minutes. Water and food were provided ad libitum inside the SA box.

The timeline of the two experiments is schematized in Fig. 2A.

### MRI acquisition

MRI acquisitions were carried out as described in^38^. In brief, we used a 9.4 T horizontal bore animal scanner (Bruker, Rheinstetten, Germany) equipped with a two elements anatomically shaped cryogenic rat surface coil cooled to 28 K. Rats were anesthetized by a gas mixture of O_2_ (50%) and air (50%) with approximately 2.5% isoflurane. Respiration rate was monitored throughout the experiment. Body temperature was maintained at 37 °C by warm water circulation and an external coil-heater and verified by a rectal thermo-sensor. T2-weighted high-resolution 3D structural data were acquired using a T2-weighted RARE sequence (Rapid Acquisition with Refocused Echoes, RARE Factor 16, TR = 1200 ms, TE = 6.25 ms) with an in-plane resolution of 0.15 mm and slice thickness of 0.3 mm. To quantify Manganese accumulation, T1 mapping images were acquired using a Rapid Acquisition with Relaxation Enhancement (RARE) sequence with variable repetition time (TE = 12.61 ms, TR = [155 250 400 800 1600 3500 6000] ms). Twenty-eight horizontal slices were planned for every subject (field of view [FOV] = 32 × 32 mm^2^, matrix size = 128 × 128, in-plane resolution = 0.25 × 0.25 mm^2^, slice thickness = 0.5 mm). The MRI scanning took approximately 90 min per animal.

### Image analysis and statistics

Behavioral characterization data were analyzed by one-way ANOVA with groups (0–1–2–3crit) as between subject factor. Behavioral data in experiment 1 were analyzed by mixed two-way ANOVA with groups (AgeControl, 0crit and 3crit) as between-subjects factor and time (MnCl_2_ treatment) as repeated measure. Infusions earned during the 24 h session in experiment 2 were analyzed by t-test for independent samples (0crit vs 3crit). Significance was conventionally set at *p* < 0.05, ANOVA was followed by Bonferroni’s post-hoc test when appropriate.

For both experiments, MRI images were realigned and normalized to a common anatomical space by SPM package. Voxel-wise T1 values were extracted by fitting the mono-exponential function $S\sim {S}_{0}\left(1-{e}^{-\frac{T1}{TR}}\right)$ using in-house built Matlab scripts. Manganese accumulation was evaluated as the T1 difference observed in the Mn^2+^ on board respect to the Mn^2+^ free acquisition ($\Delta T1=T{1}_{pre}-T{1}_{post}$), a greater ∆T1 is therefore symptom of greater Mn^2+^ accumulation and hence greater brain activity. We compared manganese accumulation in the brain of 0crit, 3crit and AgeControl rats after 1-week of drug-free period by voxel-wise one-way Analysis of Covariance (ANCOVA). Global brain manganese accumulation was considered as covariate for controlling inter-subject differences in systemic Mn^2+^ elimination. Neuronal activation during ad libitum 24 h cocaine self-administration was tested by ANCOVA corrected for brain average manganese accumulation and number of cocaine infusions earned during the session. Thresholding and correction of multiple comparisons was achieved using the threshold-free cluster enhancement (TFCE) method with a family wise error *p* of 0.05 (*p*_FWE_ < 0.05).

In addition to the voxel-wise comparisons, for the sake of clarity, reduction in T1 values were compared in a region of interest (ROI)-based fashion in 18 defined anatomical regions (depicted in Supporting Information Figure S1) which were selected for their role in mediating acute and chronic cocaine effects^47,48^. Selected regions include olfactory nucleus (ON), prefrontal cortex (PFC), insular cortex (Ins), nucleus accumbens (Acb), caudate-putamen (CPu), septum (Sept), bed nucleus of stria terminalis (BNST), globus pallidus (GP), hypothalamus (Hyp), amygdala (Amyg), habenula (Hb), hippocampus (Hc), thalamus (Thal), subthalamic nucleus (STh), substantia nigra (SN), ventral tegmental area (VTA), raphe nucleus (RNcl) and pontine nucleus (Pons). ROI-wise mean values were adjusted accordingly by the mean whole brain T1 reduction and number of injections earned. To test the effect of cocaine addict-like behavior in calcium dependent neural activity, one-way repeated-measures ANOVA was conducted. Tests were corrected for multiple comparisons using false discovery rate (${p}_{FDR}\le 0.05$). In case of significant group differences, post-hoc pairwise group comparisons were adjusted by Tukey–Kramer procedure.

## Results

### Behavioral characterization.

**Figure 1:**
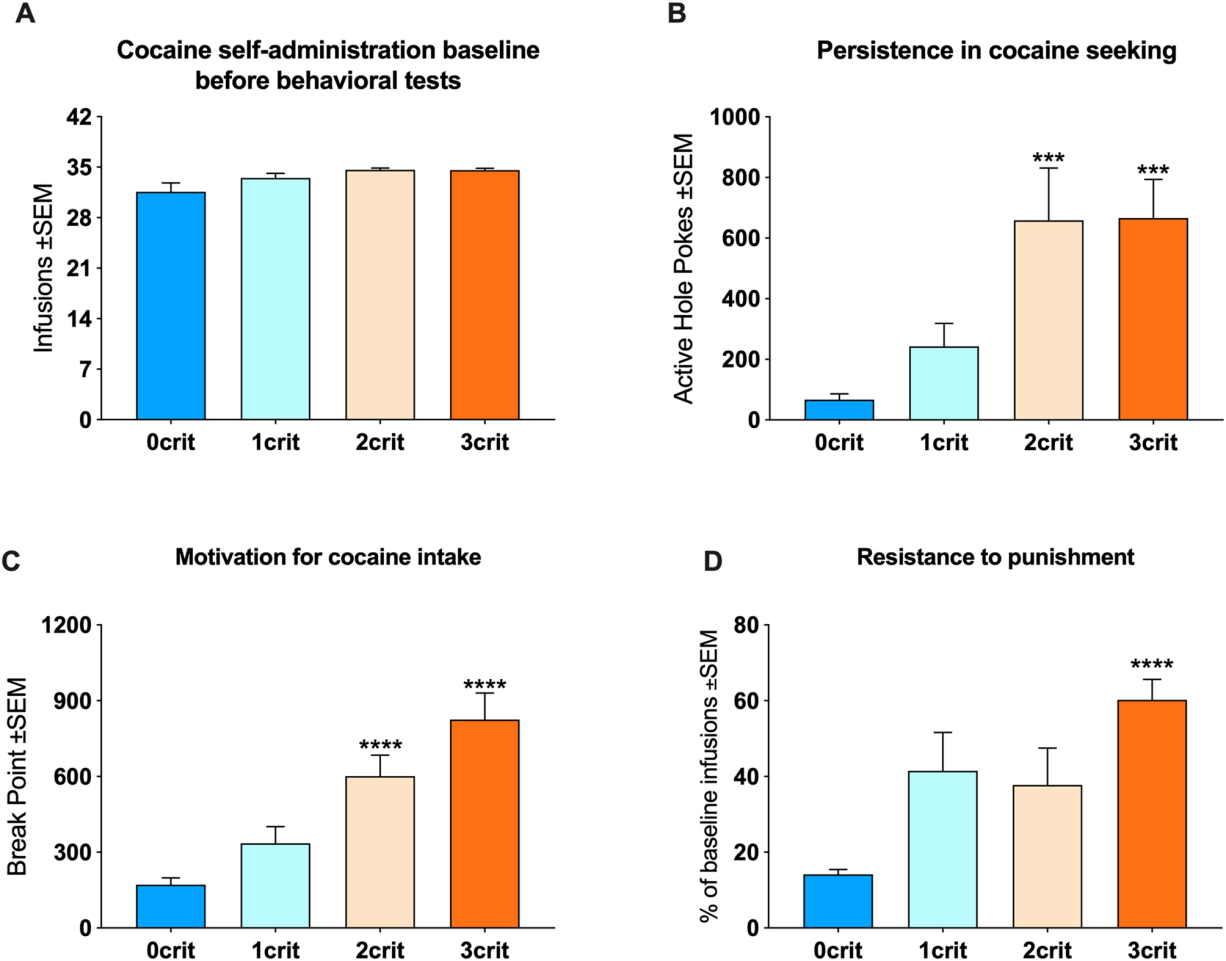
Behavioral characterization of addiction-like behavior. (**A**) 0crit, 1crit, 2crit, and 3crit rats self-administered comparable number of cocaine infusions averaged over the five days preceding characterization of addiction-like behavior. (**B**) Inability to refrain from cocaine seeking when the drug is signaled as not available, measured as persistence in drug seeking. Both 2crit and 3rit rats showed persistence in seeking, measured by non-reinforced pokes at the active hole when the drug is signaled as not available, higher than 0crit. (**C**) Motivation for cocaine expressed by the break-point reached in a progressive ratio session. Both 2crit and 3crit showed motivation for cocaine higher than 0crit. (**D**) Maintenance of cocaine self-administration despite negative consequences, expressed by resistance to punishment when drug seeking and taking is punished by electric foot-shocks. 3crit rats showed resistance to punishment higher than 0crit. Data are expressed as mean ± SEM. Statistical significance ***p < 0.001 and ****p < 0.0001 vs 0crit.

Ten rats lost catheter patency during CSA training, and therefore a final group of 51 rats were used for addiction criteria allocation and behavioral analyses. We analyzed the average of infusions earned during the last five days of CSA training before addiction criteria tests. ANOVA revealed a borderline overall effect of groups [F(3, 47) = 2.8; *p* = 0.049]; however, Bonferroni’s post-hoc test found no significant differences between groups (Fig. 1A). We also repeated the post-hoc analyses using the less conservative Newman-Keuls test, but also in this case no specific differences between groups were found. We then analyzed the three addiction-like criteria and for each of them we found an overall effect of groups: persistence in cocaine seeking [F(3, 47) = 9.5; *p* < 0.0001] (Fig. 1B); motivation for cocaine [F(3, 47) = 19.5; *p* < 0.0001] (Fig. 1C); resistance to punishment [F(3, 47) = 8.6; *p* < 0.001] (Fig. 1D). Post hoc analyses revealed that 3crit scored significantly higher than 0crit in each behavior (*p* < 0.001).

A criticism of the 0/3crit model of addiction is the use of a single punished session to assess resistance to punishment, when other works, using pseudorandom punishment protocols, demonstrated that shock-resistant and shock-sensitive rats diverge over multiple sessions^49–51^. The major difference between the pseudorandom punishment and 0/3crit models is that in the first case not every reward cycle is punished, and so rats need time to learn, whereas in the second case each reward cycle is punished twice (see methods above). This makes the presence of the punishment in the 0/3crit model easier to learn, and in fact it was demonstrated that if the single punished session is performed after only 20 CSA sessions, when addiction-like behavior is not yet developed, there is no difference between 0crit, 1crit, 2crit and 3crit rats, and each group quits self-administration^14^. Therefore, although running more punished sessions may lead to a larger divergence between 3 and 0crit rats, the 3crit model allows highlighting shock-related phenotypes with only a single shock test.

A further criticism to our experimental design is that MRI scanning was not acquired directly after the characterization of addiction-like behavior. However, there was a practical limitation in acquiring more than five scans in a single day. Therefore, all rats had some additional CSA baseline sessions before the first MRI scanning was performed. It may be argued that the behavioral difference between 0 and 3crit might have been changed during this baseline re-training. Of the three addiction criteria, we could keep monitoring the persistence in cocaine-seeking as this is recorded in every CSA session. As expected, the difference between 0 and 3crit were maintained during baseline sessions (Supplementary Fig. S2A), suggesting the stability of addict-like and non-addict-like behavior in these two groups.

### Regions of interest showing decrease in T1 relaxation time following Mn^2+^ administration

**Figure 2:**
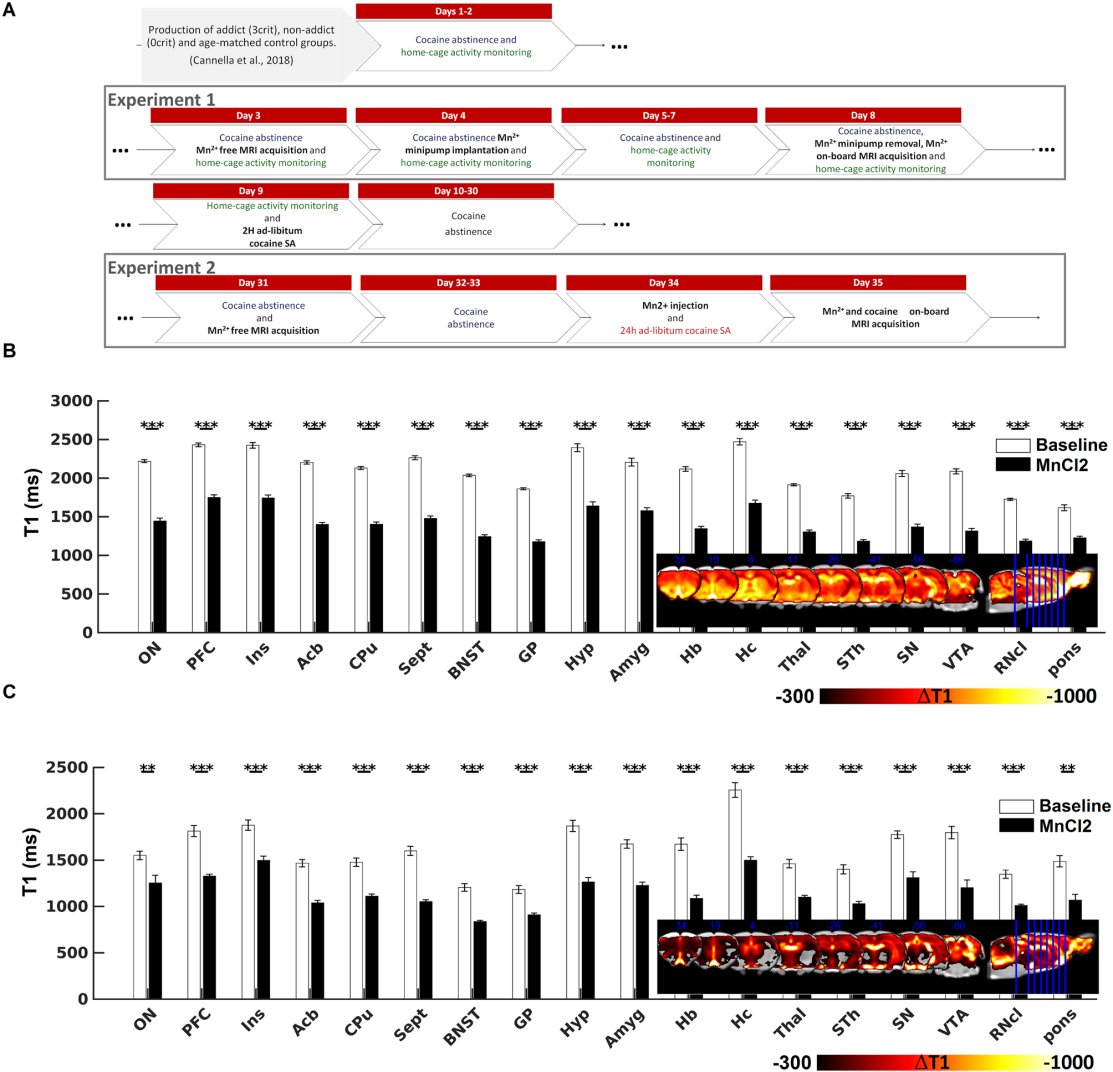
Experimental timeline and effect of systemic administration of manganese on T1 relaxation time in brain tissue in cocaine-naïve rats. (**A**) Schematic of experimental time-line. (**B–C**) comparison of T1 relaxation time in cocaine naïve rats between MnCl_2_-free and MnCl_2_-onboard acquisition following 5 days (experiment 1, **B**) and 24 h (experiment 2, **C**) of manganese uptake. Data are expressed as mean ± SEM. Statistical significance **p < 0.01, ***p < 0.001. Statistical parametric maps and bar plots were created using in-built Matlab functions. This figure was composed using CorelDRAW Standard 2020.

First we wanted to demonstrate that Mn^2+^ would accumulate in the ROIs that are relevant for mediating acute and chronic cocaine effects^47,48^. The regional distribution of Mn^2+^ accumulation is shown in Fig. 2B-C. Figure 2B shows reduced T1 relaxation time in all 18 relevant ROIs following Mn^2+^ continuous administration for five days in cocaine-naive rats in the home cage (Experiment 1). Figure 2C shows the manganese accumulation in cocaine-naive rats when 3 sub-injections in a period of 1 h were administered (Experiment 2). As expected, both MnCl_2_ administration paradigms produced a systematic reduction of T1 relaxation times. An average reduction of 32.8 ± 0.92% was observed in selected anatomical ROIs after 5 days of continuous manganese administration. A slightly less pronounced T1 relaxation time reduction was seen after repeated MnCl_2_ injections (∆T1 = 27.1 ± 1.1%). Detailed T1 reductions are showed in Supporting Information Table S1.

With both MnCl_2_ administration procedures regional distribution of T1 reduction is consistent with previous research^25,52^.

### Manganese accumulation in the brain does not affect basic behavior and cocaine reinforcement in cocaine experienced rats

**Figure 3:**
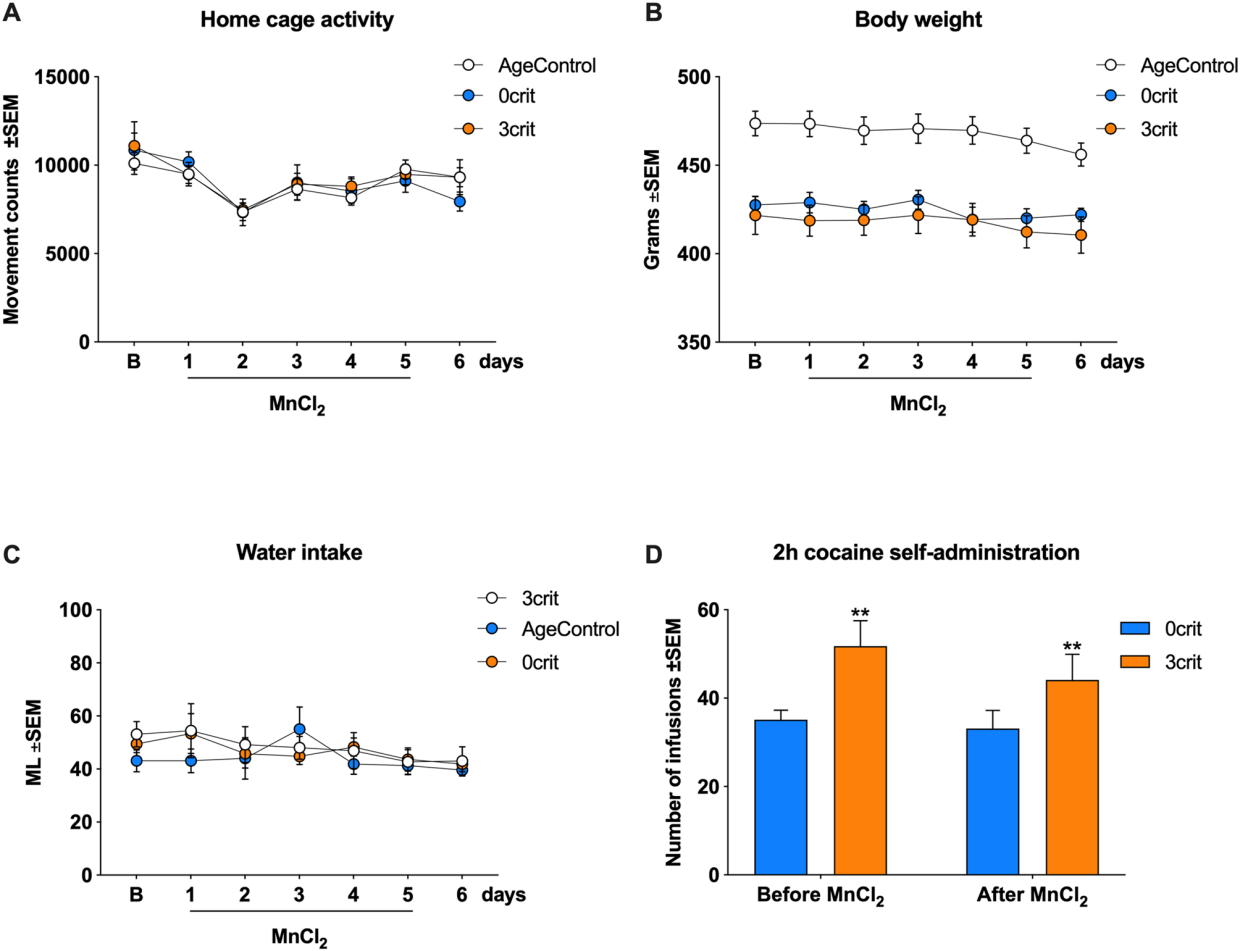
Effect of MnCl_2_ treatment on behavior and cocaine reinforcement. (**A**) The three groups of rats (0crit and 3crit rats as well as cocaine-naïve age matched control (AgeControl) groups) did not differ in baseline home cage activity. All groups had a drop in home-cage activity 24 h after implantation of infusion micro-pump—day 2—which was recovered starting from day 3. (**B**) The body weight was higher in the AgeControl group compared to 0crit and 3crit and was not affected by MnCl_2_ treatment in none of the groups. (**C**) Groups consumed comparable quantity of water. Water consumption was not affected by MnCl_2_ treatment. (**D**) Addict-like 3crit rats self-administered more cocaine infusions compared to non-addict-like 0crit rats independently from MnCl_2_ treatment. Data are expressed as mean ± SEM. Statistical significance **p < 0.01 vs 0crit.

We first evaluated whether Mn^2+^ accumulation caused health and behavioral impairments in cocaine-experienced and naïve rats. All tested rats (0crit; 3crit and AgeControl) consumed the whole 20 g/day of chow pellets provided. An analysis of home-cage activity found that all 3 groups had comparable daily activity, which dropped slightly the first 24 h after the beginning of MnCl_2_ administration and recovered at 48 h (group effect [F(2,182) = 0.26; *p* = 0.77], time effect [F(6,182) = 6.56; *p* < 0.0001], group by time interaction [F(12,182) = 0.41; *p* = 0.96], Fig. 3A). As observed in earlier studies^17,19,38,53,54^, as a consequence of cocaine exposure, average body weight was higher in the cocaine-naïve AgeControl group compared to 0crit and 3crit, but it remained stable in each group throughout the MnCl_2_ accumulation period (group effect [F(2,182) = 94.23; *p* < 0.0001], time effect [F(6,182) = 1.1; *p* = 0.36], group by time interaction [F(12,182) = 0.15; *p* = 0.99], Fig. 3B). Water intake was comparable among groups and not affected by Mn^2+^ accumulation (group effect [F(2,182) = 1.08; *p* = 0.34], time effect [F(6,182) = 1.15; *p* = 0.33], group by time interaction [F(12,182) = 0.48; *p* = 0.92], Fig. 3C). To test whether Mn^2+^ accumulation affected cocaine reinforcement in 0crit and 3crit rats, we performed two *ad-libitum* CSA sessions: one before and one after MnCl_2_ administration. As expected, addict-like rats (3crit) earned more infusions than non-addict-like rats (0crit) at both time points but importantly, the responses and reinforcers earned during these self-administration tests were similar before and after MnCl_2_ treatment (group effect [F(1,34) = 9.11; *p* = 0.0048], time effect [F(1,34) = 1.11; *p* = 0.3], group by time interaction [F(1,34) = 0.38; *p* = 0.54], Fig. 3D).

### Experiment 1: Reduced neuronal activity in cocaine addict-like rats during early cocaine abstinence evaluated by Mn^2+^ accumulation

**Figure 4:**
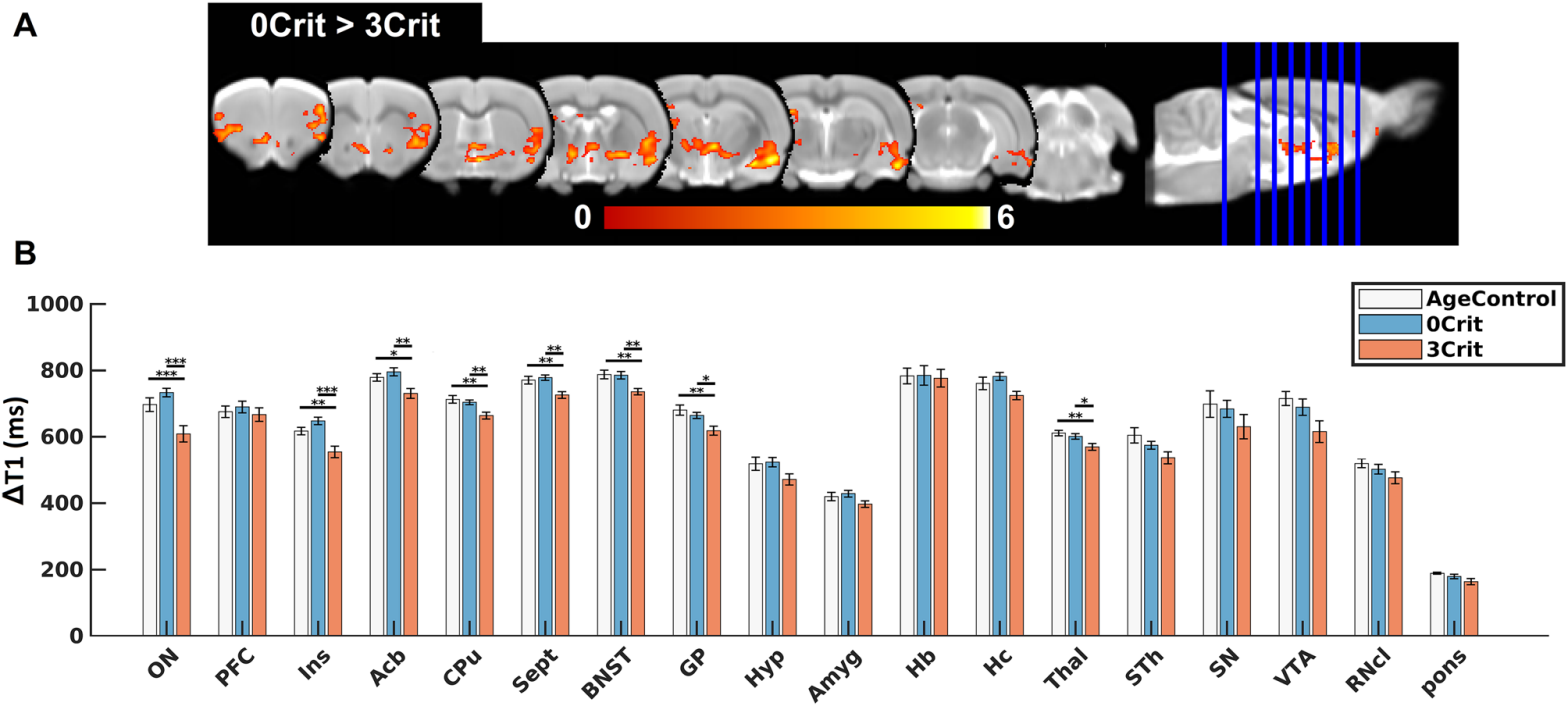
Continuous Mn^2+^ application during early cocaine revealed reduced brain activity (i.e. smaller ∆T1) in 3crit (addict-like) rats compared to age-matched naïve-control and 0crit non-addict-like rats. (**A**) Statistical anatomical maps comparing Mn^2+^ accumulation between 0 and 3crit rats. Maps were corrected for family-wise error at *p*_FWE_ < 0.05. Color-bar indicates t-stat for the comparison 0crit > 3crit. (**B**) Averaged regional Mn^2+^ uptake in 18 selected ROIs in AgeControl, 0crit and 3crit rats. Statistical tests were corrected for global Mn^2+^ accumulation. Bars represent the mean ± SEM of T1 reduction. Statistical differences: *p_FDR_ < 0.05, **p_FDR_ < 0.01, ***p_FDR_ < 0.001. Statistical parametric maps and bar plots were created using in-built Matlab functions. This figure was composed using CorelDRAW Standard 2020.

In general, we observed reduced brain activity in 3crit (addict-like) rats, while 0crit (non-addict-like) rats did not differ from cocaine-naïve AgeControl. Figure 4A shows the anatomical location of higher Mn^2+^ accumulation in 0crit rats with respect to 3crit rats superimposed in a T2w template to facilitate anatomical location of the significant results (threshold-free cluster enhancement, *p*_FWE_ < 0.05). These findings were confirmed by the ROI-based analysis. Specifically, 3crit rats showed reduced brain activity compared to both AgeControl and 0crit in several cortical and subcortical limbic areas including insula, nucleus accumbens, caudate putamen, septum, bed nucleus of the stria, globus pallidus, thalamus, and olfactory nucleus (*p*_FDR_ < 0.05). Hippocampus, subthalamic nucleus, ventral tegmental area, pontine nucleus showed also a lower activity in 3crit, which however did not reach significance threshold (*p*_FDR_ < 0.1). ROI-wise results are depicted in Fig. 4B and statistical results listed in Table 1. In addition, 0crit and 3crit rats did not differ significantly in the quantity of cocaine self-administered during the last five days of cocaine training (Supplementary Fig. S2B). This, together with the abstinence imposed before MRI acquisitions and Mn accumulation, indicates that the acute pharmacological effects of cocaine are unlikely to have influenced MEMRI signals. In summary, these results show that reduced brain activity is not simply the result of chronic cocaine intake, but it is a feature of addictive behavior.

**Table 1:** Summary of ROI wise ∆T1 and statistics for experiments 1 and 2.

ROIs	Experiment 1				Experiment 2			
	∆T1 (ms)			F_(2,26)_	∆T1 (ms)			F_(2,20)_
	AgeControl	0crit	3crit		AgeControl	0crit	3crit	
ON	696.9 ± 12.2	733.3 ± 7.5	608.9 ± 13.7	10.3**	301.9 ± 65	261.8 ± 27.0	201.3 ± 22.1	0.89
PFC	675.5 ± 10.1	690 ± 10.4	666.9 ± 11.3	0.4	494.7 ± 22.9	313.3 ± 19.2	316.4 ± 20.2	24.61***
Ins	617.2 ± 6.6	647.9 ± 6.8	554.5 ± 9.5	12.5**	379.6 ± 39.3	231.8 ± 21.6	163.4 ± 10.9	12.05***
Acb	778.9 ± 6.6	795.6 ± 7.1	730.7 ± 8.3	6.8*	439.6 ± 33.2	311.0 ± 21.0	291.0 ± 8.9	9.07**
CPu	712.9 ± 6.8	704 ± 4.1	664.1 ± 5.8	6.8*	374.9 ± 10.2	217.3 ± 13.7	211.8 ± 11.8	58.36***
Sept	770.9 ± 6.8	778.4 ± 4.7	726.1 ± 5.7	7.7*	541.3 ± 13.0	415.5 ± 14.3	402.4 ± 16.3	29.10***
BNST	787.7 ± 7.7	785.4 ± 6.7	735.9 ± 5.3	6.2*	367.9 ± 15.4	239.3 ± 12.1	244.8 ± 12.2	28.73***
GP	680.6 ± 9	664.5 ± 5.4	618.5 ± 7.6	5.9*	289.2 ± 11.1	161.2 ± 14.5	173.0 ± 12.8	30.89***
Hyp	518.7 ± 11.7	523.6 ± 8.2	471.6 ± 9.5	2.7	382.2 ± 21.4	289.0 ± 14.5	242.9 ± 15.4	13.82***
Amyg	420 ± 7.4	428.6 ± 5.9	397.3 ± 5.4	2.1	278.3 ± 13.1	182.2 ± 11.9	150.7 ± 7.6	28.07***
Hb	783.5 ± 13.8	785 ± 17.3	776.5 ± 14.9	0	590.6 ± 17.4	365.7 ± 27.8	335.5 ± 9.5	38.78***
Hc	760.8 ± 11.1	781.9 ± 7	724.6 ± 7	3.7	714.4 ± 17.5	510.2 ± 16.8	486.3 ± 10.1	56.12***
Thal	611.1 ± 4.9	601 ± 4.8	569.4 ± 5.7	5.8*	378.3 ± 8.3	225.4 ± 8.6	225.4 ± 9.2	107.15***
STh	604.6 ± 13.5	574.6 ± 6.9	536.7 ± 10	3.4	367.0 ± 40.6	272.9 ± 18.6	218.3 ± 12.3	5.49*
SN	698.6 ± 23.5	684.2 ± 15.1	630.5 ± 20.4	1	546.2 ± 63.6	370.4 ± 30.4	365.5 ± 23.6	4.69*
VTA	715.3 ± 12.3	689.2 ± 14.5	615.6 ± 18.2	3.8	608.2 ± 61.6	354.8 ± 24.5	332.0 ± 41.6	10.74***
RNcl	519.4 ± 7.5	502.3 ± 8.6	476.7 ± 10	2	331.2 ± 14.1	213.9 ± 12.5	237.1 ± 13.2	23.10***
pons	188.9 ± 1.7	179.1 ± 3.9	163.7 ± 5.1	3.7	130.7 ± 7.8	69.6 ± 6.2	74.9 ± 12.6	18.74***

Averaged regional T1 reduction and F values of ROI-wise statistics in 18 selected ROIs in AgeControl, 0crit and 3crit rats after 5 days of continuous Mn^2+^ loading at early abstinence (**Experiment 1**) and 24 h cocaine bingeing (**Experiment 2**). Statistical tests were corrected for global Mn^2+^ accumulation. ∆T1 are reported as mean ± SEM. Statistical differences: *p_FDR_ < 0.05, **p_FDR_ < 0.01, ***p_FDR_ < 0.001.

### Experiment 2: Cocaine bingeing reduces neuronal activity in 0crit and 3crit animals

**Figure 5:**
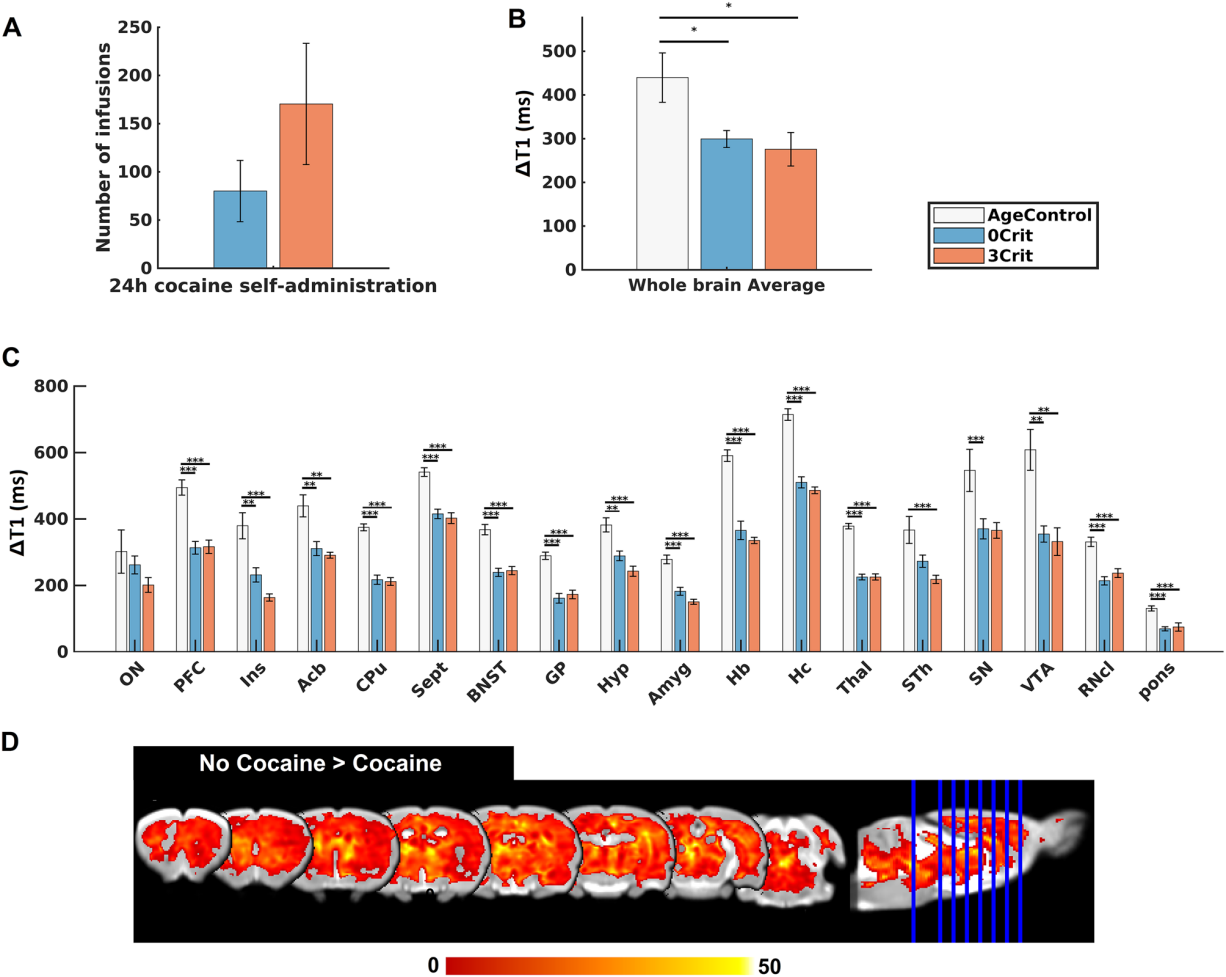
Acute Mn^2+^ accumulation during 24 h cocaine bingeing. Reduced brain activity (i.e. smaller ∆T1) is seen in both 0crit and 3crit rats compared to cocaine-naïve Age-Control rats. (**A**) During 24 h self-administration 3crit rats tend to self-administer a higher number of cocaine infusions compared to 0crit. (**B**) Average whole brain uptake was reduced in both 0crit and 3crit rats compared to AgeControl. (**C**) Similarly to whole brain activity, also within specific ROIs there was a reduction in brain activity in cocaine exposed rats (0crit and 3crit) independently from addiction-like phenotype. (**D**) Statistical anatomical maps comparing brain activity between rats subjected to 24 h cocaine self-administration (0crit and 3crit matched) and cocaine naïve age-matched control group subjected to the sham protocol. Statistical tests were corrected for global Mn^2+^ accumulation and cocaine effect. Maps were corrected for family-wise error at *p*_FWE_ < 0.05. Color-bar indicates t-stat for the comparison No Cocaine > Cocaine. Bars represent the mean ± SEM of T1 reduction. Statistical differences: **p_FDR_ < 0.01, ***p_FDR_ < 0.001. Statistical parametric maps and bar plots were created using in-built Matlab functions. This figure was composed using CorelDRAW Standard 2020.

Of the ten 0crit and nine 3crit rats entering the study, one 0crit and one 3crit lost catheter patency before they entered the 24 h CSA session, and three 3crit were found dead in the self-administration box after the session. Therefore, Mn^2+^ onboard MRI was acquired only in nine 0crit and five 3crit rats. 3crit rats self-administered a significantly higher number of cocaine infusions than 0crit [t(15) = 2.24; *p* = 0.04], but after removing the rats found dead after the 24 h CSA session and considering only those subjects entering Mn^2+^ onboard MRI acquisition, the difference was no longer statistically significant [t(12) = 1.44; *p* = 0.17] (Fig. 5A). However, this not-statically-significant difference may well derive from a type II error given the low sample sizes. Therefore, we estimated Cohen’s effect size and found a value of d = 0.803, conventionally interpreted as a large effect size^55^ and therefore indicating that 3crit rats indeed consumed more cocaine than 0crit rats. Whole brain activity (i.e. Mn^2+^ accumulation) was extensively decreased in both 0crit and 3crit compared to age matched cocaine-naïve control rats [F_(2,22)_ = 4.67, *p* < 0.05] (Fig. 5B). In order to rule out confounding effects, statistical analyses were adjusted with earned cocaine infusions and whole brain activity as described in the methods section. Thus, in each of the specific ROIs tested, except the olfactory nucleus [F_(2,20)_ = 0.89, *p*_FDR_ > 0.05], a significant group effect was observed (*p*_FDR_ < 0.05). 0crit and 3crit did not differ from each other and both showed lower brain activity compared to age matched cocaine-naïve controls (Fig. 5C). Detailed descriptive statistics and group effects are shown in Table 1. Since 0crit and 3crit did not differ from each other either in whole brain or in specific ROIs activity, we also matched these two groups in a single “cocaine” group and compared their brain activity with age-controls by voxel-wise analysis and showed reduced activity in the whole brain, with the exception of the olfactory bulb and part of the cerebellum (threshold-free cluster enhancement, *p*_FWE_ < 0.05) (Fig. 5D). These data show that irrespective of being addict-like or non-addict-like to the drug, acute cocaine bingeing strongly reduced neuronal activity.

## Discussion

Here, using a MEMRI approach, we demonstrated that addict-like rats exhibit reduced neuronal activity compared to age-matched cocaine-naïve controls during the first week of abstinence from cocaine. In contrast, cocaine-experienced rats that did not develop addict-like behavior (0crit) maintained their brain activity at a level comparable to cocaine-naïve control rats. We also used MEMRI to evaluate brain activity during cocaine bingeing and found a general reduction of brain activity in cocaine experienced rats independently of an addiction-like phenotype. The effects observed in both experiments may reflect both Mn local absorption and diffusion, as manganese is known to diffuse along axons^56,57^.

Although manganese is an essential heavy metal, it has neurotoxic effect following excessive exposure^58^. Therefore, we also tested whether our Mn^2+^ application protocols affected general behavior and cocaine reinforcement. Under our experimental conditions, Mn^2+^ had no or negligible effect on rats’ behavior, general health state or on cocaine reinforcement. This indicates that the differences observed in terms of neuronal activity can be attributed to individual differences in addictive behavior.

Compared to our recent report using FDG-PET, the results presented here provide a different perspective on brain activity associated with addiction vulnerability and resilience, broadening the quality and quantity of information. Our results corroborate the generally reduced cortical activity found in 3crit compared to cocaine-naïve rats, while 0crit had an activity level similar to controls^19^. However, our MEMRI approach revealed a much broader effect, including several cortices and sub-cortical nuclei. This broader effect may be due to a longer exposure (5 days) to the MRI enhancer Mn^2+^ compared to the relatively short exposure to the ^18^FDG used in our PET study (40 min)^19^, and/or to handling procedures associated with FDG administration and preparation for scanning, as MEMRI rats were left undisturbed for most of the five-days Mn-uptake period except for daily monitoring of bodyweight, food and water consumption. In addition to general agreement between the studies, we also observed differences between FDG and MEMRI results. For instance, while the current study found no group differences in ∆T1 in the PFC and reduced ∆T1 (i.e. activity) in the CPu of 3crit, with ^18^FDG we reported increased glucose uptake in both PFC and CPu of 0crit^19^. This suggests that ^18^FDG and MEMRI may capture different aspects of brain activity, in which ^18^FDG gives a measure of the energy consumption not exclusively associated with neural transmission while MEMRI reflects Ca^2+^ influx and hence neuronal depolarization.

We observed decreased Mn^2+^ accumulation in the Acb and CPu of 3crit compared to cocaine naïve control and 0crit rats while no group differences were observed in the PFC. This indicates an addiction-specific decrease in neuronal activity in the mesocorticolimbic circuit, a brain network responsible for the evaluation of rewarding and aversive stimuli to promote goal-directed behavior, the functionality of which is impaired in addiction^35,59,60^. Similarly, we found an addiction-specific decreased activity in the insula, a brain site that mediates interoceptive and emotional aspects of reward that is hypofunctional in addicts^33^. As a Ca^2+^ analogue, Mn^2+^ enters the cells through calcium channels and is taken up upon neuronal depolarization, and thus gives a quantitative measure of changes in calcium channels activity^61^. Exposure to cocaine impairs the modulation of Ca^2+^ homeostasis by dopamine receptors, leading to a net decrease in Ca^2+^ influx^62^, which is also associated with decreased neuronal excitability^63^. Therefore, reduced Mn^2+^ uptake in 3crit rats may indicate a chronic hypo-functionality of the mesocorticolimbic system in these rats. Interestingly, a cocaine-induced reduction of Ca^2+^ influx is associated with reduced dopamine receptor 2 (D2R) expression, a marker of psychostimulant addiction^64–66^, and impaired ability of D2R to modulate calcium homeostasis^62^.

It remains to be determined whether the group differences observed developed during CSA training or were constitutively expressed by 3crit, 0crit and cocaine naïve control groups. A dedicated longitudinal study will be necessary to clear this point. Nonetheless, previous studies have demonstrated that the neurobiological and behavioral underpinnings of addict-like and non-addict-like behavior in 3crit and 0crit respectively develop during CSA training^14,15,40,41^, though predisposing traits such us high impulsivity and novelty seeking exist before any cocaine experience^39,42^. Therefore, although specific evidence still needs to be provided, it can be speculated that the effects observed were a consequence of cocaine experience and 0crit and 3crit showed different adaptation to cocaine exposure.

This work was conducted exclusively in male rats. A previous study demonstrated that female rats allowed to self-administer ad libitum amount of cocaine in long access sessions self-administered higher amount of cocaine and showed higher escalation compared to males^67^. This indicate that findings relevant to cocaine dependence in male rats do not necessarily reproduce in female rats.

In sum, our present MEMRI experiments indicate a reduction in brain activity in 3crit rats during early cocaine withdrawal. This is in line with human data reporting brain hypo-functionality in cocaine addicts^4,5,20,21,30,68,69^. Cerebral hypoactivity has also been suggested as a biomarker of cocaine addiction as it is associated with reduced ability to experience reward^6,7^ and control impulses^9,10^. It may also predict relapse risk^11–13^. Our translational neuroimaging study further confirms the presence of neuronal hypoactivity in cocaine-addicted subjects. However, the additional value of this dataset to the addiction field is the extension of our clinical knowledge by the conclusions that reduced neuronal activity in addicted individuals is associated with core symptoms of addictive behavior, is associated with the development of compulsive use, and is not simply the result of chronic cocaine intake. In other words, since 0crit and 3crit rats consumed comparable amounts of cocaine during their lifespan^38^ (Fig. 1A) and right before experiment 1 (Supplementary Figure S2B), the MEMRI effects observed in the 3crit animals are not associated with the quantity of cocaine consumed but rather derive from their innate vulnerability to addict-like behavior. In contrast, 0crit rats have a profile allowing them to resist or counteract cocaine-induced adaptations^40,41^. These conclusions (i) emphasize the notion that preclinical screening should devote more attention to individual variability in drug response and (ii) supports the idea that studying cerebral hypoactivity is a way to pursue biomarkers of cocaine addiction.

In the second experiment we tested brain activation during a 24 h cocaine self-administration binge. Our data indicate that brain activity during bingeing does not differ between 0 and 3crit rats, possibly because of the relatively rapid development of tolerance^70^ that is reflected in reduced brain activity^71^. This is in line with our FDG-PET study demonstrating that 0crit and 3crit did not differ in metabolic response to an acute cocaine challenge^19^, and is consistent with the interpretation that differences in addictive-like behavior between 0 and 3crit rats is independent from the primary reinforcing effect of cocaine^15^. Previous studies have also used MEMRI to examine cocaine-induced brain activation. Lu and co-workers tested the effect of an acute cocaine injection in naïve rats and found increased activity brain-wide^24^; others have used MEMRI to study cocaine-induced behavioral sensitization^26^ and sex differences in cocaine-induced brain activation^25^. All of these previous studies have used either acute cocaine treatment or relatively short repeated treatments, and cocaine experience was always non-contingently (i.e. operator) administered. In contrast, for the first time we report here on brain activity by MEMRI in rats that self-administered cocaine in a binge session and, more importantly, in addict-like and non-addict-like rats that were subjected to prolonged (> 50 days) cocaine self-administration training^38^, demonstrating that in these conditions cocaine experience produces a brain-wide decrease in neuronal activity. Since Mn reaches the brain through the vascular system, an alternative interpretation of experiment 2 results could be that the decreased Mn^2+^ uptake found in cocaine-experienced rats may reflect reduced cerebral blood flow (CBF) induced by cocaine^72–75^ rather than decreased neuronal activity due to cocaine bingeing. However, although the contribution of lower blood supply in reduced Mn uptake by cocaine-experienced rats cannot be excluded, previous works reported increased neuronal Ca^2+^ influx^76^ and activity^77^ coupled with a cocaine-induced CBF reduction, and increased Mn^2+^ uptake associated with reduced CBF^56^. In addition, it should be noticed that we observed no cocaine effect in the olfactory bulb (Fig. 5D), an area not included among the addiction-related ROIs that could therefore serve as negative control, as we would have seen a reduced Mn uptake in this area if this was solely due to reduced blood supply. A possible concern with this interpretation could be that the olfactory bulb may receive limited vascularization and hence a lower Mn supply compared to other areas; however, CBF in the olfactory bulb is similar to the rest of the brain^78^ and Mn uptake is in line with other areas (Fig. 2B,C). In summary, although a possible limitation associated with the effects of cocaine on blood supply and ROIs vascularization should be taken into account, our results indicate that during the cocaine binge there is a reduction in neuronal activity that is similar between 0 and 3crit rats, suggesting that this reduction may be a marker of cocaine bingeing independent of an addiction-like phenotype.

MEMRI studies sometime use blood–brain-barrier (BBB) disruption to induce a homogeneous distribution of Mn^24^, which otherwise may be less absorbed in areas distant from the ventricles^45^. Here we decided not to disrupt the BBB to preserve rats’ health and observe differences in their physiological conditions. However, it is unlikely that the low absorption rate is responsible for the lack of group differences observed in certain areas, as we have also shown that Mn is readily absorbed in the whole brain (Fig. 2B,C).

We found reduced brain activity in 3crit rats in both experiment-1 and experiment-2, and therefore one interpretation is that the two experiments reflect the same phenomenon and the 3crit rats simply have a slower rate of recovery. However, it should be noted that in experiment-1 the Mn-free and Mn-onboard MRI scans were acquired two and seven days into abstinence, respectively, and Mn was loaded between the two acquisitions. Conversely, in experiment-2, Mn was loaded contingently with 24 hour-binge CSA. Considering that prior to experiment-1 rats were subjected to short-access sessions with a 35-infusion limit and that in this condition cocaine is completely cleared from brain and blood within 4–5 h^79–81^, we suggest that reduced activity in 3crit rats observed in experiment-1 reflects an abstinence condition in a cocaine-free state, while in experiment-2 reflects an acute pharmacological effect of cocaine.

In conclusion, two main messages emerge from the data reported here. First, although 0crit and 3crit rats had a comparable life-long level of cocaine exposure^38^, 0crit (non-addict-like rats) show brain activity similar to cocaine-naïve age matched control rats, while 3crit (addict-like rats) show reduced activity. Second, cocaine bingeing affects brain activity similarly in addict-like (3crit) and non-addict-like (0crit) rats. Together these findings indicate that although drug exposure is a necessary condition to develop addiction, there are individual predisposing traits that determine the development of addictive behavior. This should instruct preclinical researchers working on treatment development to pay attention to individual-based differences. More importantly, our study provides critical translational information for the clinical situation and supports the idea that attention should be focused on cerebral hypoactivity during abstinence when studying biomarkers for cocaine addiction.

## Supplementary information

Supplementary Information.
