# Supplementary material for: Cocaine addicted rats show reduced neural activity as revealed by manganese-enhanced MRI
Source: Sci Rep. 2020 Nov 9;10:19353. doi: 10.1038/s41598-020-76182-3 (PMC7653042; doi:10.1038/s41598-020-76182-3)
Supplement: Supplementary file 1 — Supplementary Information. [file 41598_2020_76182_MOESM1_ESM.pdf]

**Supplementary Information for:**

# Cocaine addicted rats show reduced neural activity as revealed by manganese-enhanced MRI

Nazzareno Cannella[1,§,*], Alejandro Cosa-Linan[1,§], Tatiane Takahashi[1], Wolfgang Weber-Fahr[2], Rainer Spanagel[1]

[1] Institute of Psychopharmacology, Central Institute of Mental Health, Medical Faculty Mannheim, Heidelberg University, Germany

[2] Research Group Translational Imaging, Department of Neuroimaging, Central Institute of Mental Health, Medical Faculty Mannheim, Heidelberg University, Germany

[§] These authors contributed equally

| ROI | Experiment1 | | Experiment2 | |
|---|---|---|---|---|
| | ΔT1 | t-stat | ΔT1 | t-stat |
| ON | 34.9±1.7% | 19.61*** | 19.1±4.8% | 3.69** |
| PFC | 28±1.4% | 18.36*** | 26.0±3.1% | 6.28*** |
| Ins | 28.1±1.2% | 20.92*** | 19.8±2.9% | 5.80*** |
| Acb | 36.4±1.4% | 23.33*** | 28.9±1.9% | 11.76*** |
| CPu | 34.1±1.0% | 33.22*** | 24.0±3.0% | 6.14*** |
| Sept | 34.7±1.4% | 21.94*** | 33.7±2.2% | 10.06*** |
| BNST | 38.9±1.1% | 31.19*** | 29.8±2.82% | 7.62*** |
| GP | 36.7±1.2% | 28.79*** | 22.3±2.6% | 6.46*** |
| Hyp | 31.4±2.3% | 12.24*** | 32.1±2.4% | 10.70*** |
| Amyg | 28.3±1.7% | 13.36*** | 26.1±3.1% | 6.83*** |
| Hb | 36.5±1.2% | 27.74*** | 34.4±2.9% | 8.30*** |
| Hc | 32.0±1.8% | 15.10*** | 32.9±2.82% | 8.05*** |
| Thal | 31.8±1.1% | 26.99*** | 24.0±3.0% | 6.08*** |
| STh | 33.0±1.7% | 15.32*** | 25.8±3.0% | 6.69*** |
| SN | 33.3±2.3% | 12.41*** | 26.0±3.7% | 6.62*** |
| VTA | 37.0±1.6% | 21.01*** | 32.3±5.4% | 5.23*** |
| RNcl | 31.1±1.5% | 19.03*** | 24.3±2.9% | 6.33*** |
| pons | 23.6±2.8% | 7.86*** | 26.9±5.5% | 4.94** |

**Table S1.** T1 reduction was observed brain-wide in cocaine naïve rats after 5 days (Experiment 1) and 24 h (Experiment 2) after continuous or acute manganese administration, respectively. Data are expressed as mean ± SEM; **$p_{FDR}<0.01$, ***$p_{FDR}<0.001$.

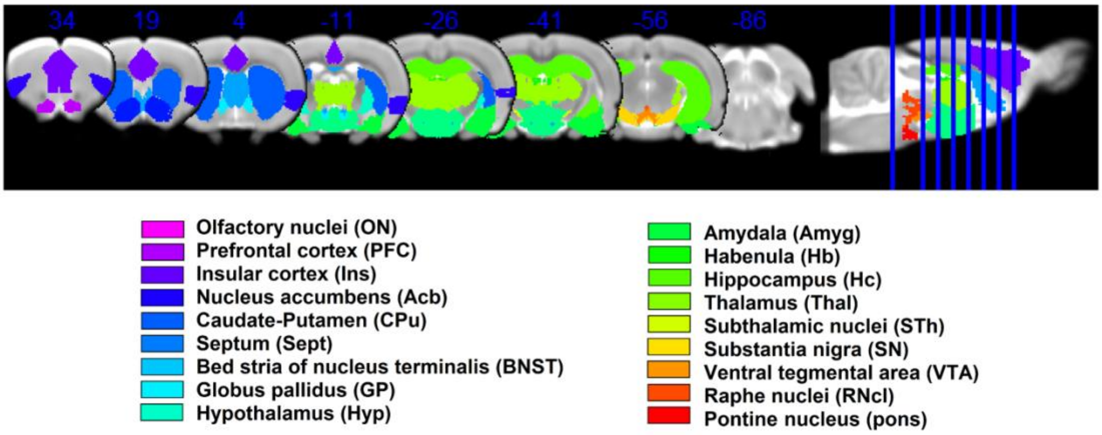


**Figure S1.** MRI atlas showing the ROIs (derived from Noori et al., 2012; 2017[1,2]) superimposed in a T2-weighted template. Statistical parametric maps were created using in-built Matlab functions. This figure was composed using CorelDRAW Standard 2020.

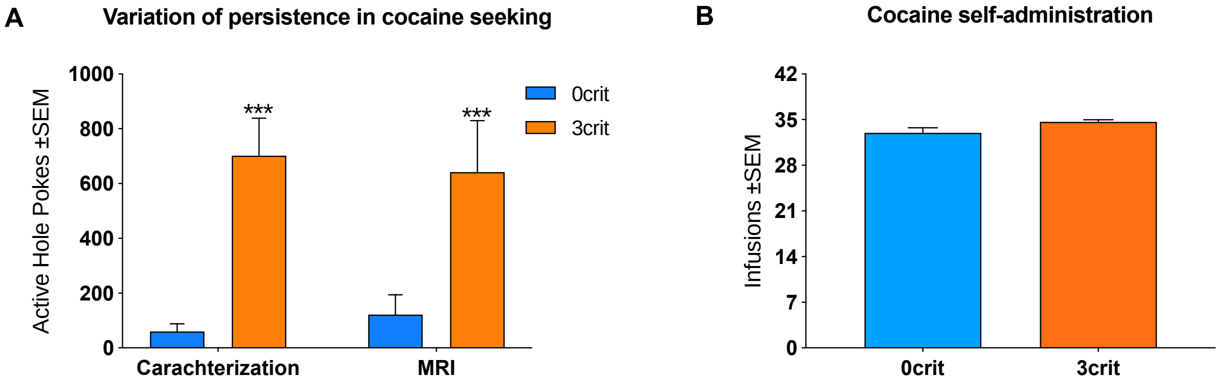


**Figure S2. A)** Persistence in cocaine seeking expressed by 0crit and 3crit rat at the time of characterization of addiction-like behavior (Characterization) and during the last five baseline CSA sessions before the first MRI acquisition were compared. ANOVA revealed an effect of group [F(1,17)=17.2; p=0.0007] but no effect of time [F(1,17)=0.0002; p=0.99] and time by group interaction [F(1,17)=0.5; p=0.5], indicating that groups difference in persistence in cocaine seeking

were maintained over time. **B)** Infusions earned in average during the last five days of cocaine self-administration baseline (i.e. prior to experiment 1) by 0crit and 3crit did not differ significantly [t(17)=1.9; p=0.075].

## REFERENCE


1 Noori, H. R. *et al.* A multiscale cerebral neurochemical connectome of the rat brain. *PLoS Biol* **15**, e2002612, doi:10.1371/journal.pbio.2002612 (2017).

2 Noori, H. R., Spanagel, R. & Hansson, A. C. Neurocircuitry for modeling drug effects. *Addict Biol* **17**, 827-864, doi:10.1111/j.1369-1600.2012.00485.x (2012).
